# Ovarian status modulates cardiovascular autonomic control and oxidative stress in target organs

**DOI:** 10.1186/s13293-020-00290-y

**Published:** 2020-04-07

**Authors:** Maycon Junior Ferreira, Iris Callado Sanches, Luciana Jorge, Susana Francisca Llesuy, Maria Cláudia Irigoyen, Kátia De Angelis

**Affiliations:** 1 Physiology Exercise Laboratory, Department of Physiology, Federal University of Sao Paulo (UNIFESP), Sao Paulo, Brazil; 2 Human Movement Laboratory, Sao Judas Tadeu University (USJT), Sao Paulo, SP Brazil; 3 Hypertension Unit, Heart Institute, University of Sao Paulo (USP), Sao Paulo, Brazil; 4 University Institute of Italian Hospital, Italian Hospital of Buenos Aires (HIBA), Buenos Aires, Argentina; 5 Laboratory of Translational Physiology, Nove de Julho University (UNINOVE), Sao Paulo, SP Brazil

**Keywords:** Ovarian hormones, Menopause, Baroreflex sensitivity, Oxidative stress

## Abstract

Studies have presented conflicting findings regarding the association between both fluctuation and deprivation of ovarian hormones and cardiovascular autonomic modulation and oxidative stress and their potential impact on resting arterial pressure (AP) and cardiovascular risk. This study aimed to assess cardiovascular autonomic modulation, baroreflex sensitivity (BRS), and oxidative stress in male rats (M) and in female rats during ovulatory (FOV) and non-ovulatory phases (FNOV) of the estrous cycle and after deprivation of ovarian hormones (FO). Direct AP was recorded, and BRS was assessed by using increasing doses of phenylephrine and sodium nitroprusside. AP and heart rate variability were assessed by spectral analysis. Oxidative stress profile was evaluated in cardiac, renal, and muscle tissues. In females, the ovulatory phase and ovarian hormone deprivation induced an increase in AP (FOV and FO ~ 9 mmHg) when compared to the non-ovulatory phase. Ovariectomy promoted increased cardiac sympathovagal balance (~ 17–37%) when compared to other groups. Both FOV and FO groups presented impaired BRS, associated with higher AP variability. In general, antioxidant capacity was higher in the FNOV than in the M group. Ovarian hormone deprivation induced a decrease in catalase activity in cardiac and renal tissues and an increase in lipid peroxidation in all tissues analyzed. Positive correlations (*p* < 0.05) were found between vascular sympathetic modulation and lipid peroxidation in cardiac (*r* = 0.60), renal (*r* = 0.60), and muscle (*r* = 0.57) tissues. In conclusion, both oscillation and deprivation of ovarian hormones play an important role in cardiovascular autonomic control and oxidative stress profile in target organs, which is reflected in AP changes.

## Introduction

In males and females, the prevalence of cardiovascular diseases (CVD) increases with age [1], regardless of sex. Historically, however, women in reproductive age have been found to be less affected by cardiovascular events than men [1]. In addition, it is well established that gender-specific/gender-related risk *factors for CVD,* such as menopause, strongly impact female health. *Postmenopausal women* also have a higher *risk* of *CVD* than males of the same age [2, 3].

Ovarian hormone concentrations fluctuate during the menstrual cycle in order to fulfill their biological functions. In this context, it is suggested that estrogen and progesterone fluctuations change arterial pressure (AP) [4]. More particularly, several studies have shown that the hormonal oscillations during the menstrual cycle modulate AP and baroreflex control. Minson et al*.* [5] have observed increased sympathetic baroreflex sensitivity in the presence of elevated levels of ovarian hormones during the menstrual cycle in young women. However, no differences have been found between early (i.e., low estrogen and low progesterone) and late follicular (high estrogen and low progesterone) menstrual periods regarding increases in both HR and AP. Also, these early/late phases do not seem to play a role in the reduction of spontaneous cardiac baroreflex sensitivity by muscle metaboreflex activation in young women [6]. In addition, we have demonstrated impaired arterial pressure variability and baroreflex sensitivity, which were associated with AP increase after deprivation of ovarian hormones in rats [7–9].

Importantly, studies focusing on impairments in HR and AP variability and in baroreflex control have found them to be strongly associated with end-organ damage and increased mortality [10, 11]. Oxidative stress has been implicated in the genesis and progression of cardiovascular disease, while the reduction of estrogens may lead to oxidative stress [12], endothelial dysfunction, and increased AP [13]. Massafra et al*.* [14] have found antioxidant variation during the phases of the menstrual cycle and observed an increase in the activity of glutathione peroxidase (GPx) in the phases of higher concentration of estrogen. Other studies have observed an improvement in antioxidant potential as a response to estrogen regulation [15], which contributes to a greater availability of nitric oxide [16]. Previous studies from our group have demonstrated increased cardiac oxidative stress associated with baroreflex sensitivity dysfunction and AP increase in ovariectomized rats [8]^.^

Although some studies have suggested that both fluctuation and deprivation of ovarian hormones may impact the regulatory mechanisms of AP and while others have pointed to the role of oxidative stress status in target organs, these findings remain largely controversial and poorly understood. Thus, unraveling the effects of ovarian hormones on autonomic function and oxidative stress in target organs is important for understanding sex differences in cardiovascular health and disease. In this study, we aimed to evaluate the effects of ovarian hormones in female rats on cardiovascular autonomic modulation and baroreflex sensitivity, as well as on the profile of target organ oxidative stress when compared to male rats. We expected that in addition to the direct impact on cardiovascular autonomic control of circulation, the oscillation of ovarian hormones induces changes in pro-oxidant/antioxidant defense in target organs, resulting in changes in AP, while deprivation of ovarian hormone would promote more pronounced alterations. Additionally, we may also suggest a potential association between systolic arterial pressure variability and oxidative stress damage in target organs.

## Materials and methods

Male and female Wistar rats (200–250 g) were obtained from Sao Judas Tadeu University (Sao Paulo, SP, Brazil). The rats were distributed into four experimental groups (*n* = 7 rats each group): male (M), female in the non-ovulatory phase of the estrous cycle (FNOV), female in the ovulatory phase of the estrous cycle (FOV), and ovariectomized females (FO). They were given standard laboratory chow and tap water ad libitum. The rats were maintained in a temperature (22–24°)-controlled room under a 12/12-h dark/light cycle. All procedures and protocols used were previously approved by the Ethics Committee of Sao Judas Tadeu University (protocol 01/2008).

### Determination of the estrous cycle phases

The characterization of each phase of the estrous cycle in female rats was based on the three types of cells in vaginal secretion: epithelial, corneal, and leukocytes [17]. Vaginal secretion was collected using a plastic pipette containing 10 μL of saline solution introduced superficially into the vagina of the rat. This drop of saline solution was collected and placed in a glass blade for observation in an optical microscope (magnified by × 400). Measurement was performed in the ovulatory (estrus) and non-ovulatory (diestrus) phases of the estrous cycle.

### Ovariectomy

At 90 days old, after confirmation of reproductive life, the female rats were anesthetized with ketamine (50 mg/kg, Ketalar 10% or 100 mg/mL, Park-Davis) and xylazine (12 mg/kg, 2% Rompum or 20 mg/mL, Bayer) and placed in dorsal decubitus for a small incision (1 cm) in parallel with the body line in the skin and muscles in the lower third in the abdominal region. The ovaries were located, and the ligation of the oviducts was performed, including the blood vessels. The oviducts were sectioned, and the ovaries removed. The muscles and skin were sutured, and a dose of antibiotic (40,000 U/kg penicillin G procaine IM) was administered [8]. Cardiovascular evaluations were performed 60 days after ovariectomy.

### Estrogen levels

In this study, the estrogen concentration, measured by immunoassay, was higher in the FNOV group (31.3 ± 2.7 pg/mL) as compared to the FOV group (12.8 ± 1.7 pg/mL), and it was non-detectable in the M and FO groups.

### Cardiovascular assessment

On the last day of the protocol, rats were anesthetized with ketamine (80 mg/kg) and xylazine (12 mg/kg) and placed in the supine position. A small incision was made near the neck to implant two polyethylene-tipped Tygon cannulas: into the carotid artery toward the left ventricle for direct BP recording and in the jugular vein for drug infusion, respectively. The cannulas were passed subcutaneously, externalized on the back of the cervical region, and fixed with cotton thread on the skin. Analgesia was administered after the surgery.

Rats received food and water ad libitum and were studied after catheter placement; they remained conscious and awake in their cages during the hemodynamic measurements. The arterial cannula was connected to a transducer (Blood Pressure XDCR, Kent Scientific), and blood pressure signals were recorded over a 30-min period using a microcomputer equipped with an analog-to-digital converter (Windaq, 2-Hz sampling frequency, Dataq Instruments). The recorded data were analyzed on a beat-to-beat basis to quantify changes in systolic (SAP), diastolic (DAP), mean AP (MAP), and HR [7–9].

### Cardiovascular autonomic assessment

After baseline AP measurement, baroreflex sensitivity was assessed by using increasing doses of phenylephrine (0.5 to 2.0 g/mL) and sodium nitroprusside (5 to 20 g/mL) given as sequential bolus injections (0.1 mL) to produce AP rise and fall responses ranging from 5 to 40 mmHg each. A 3- to 5-min interval between doses was necessary for AP to return to baseline. Peak increases or decreases in MAP after phenylephrine or sodium nitroprusside injection and the corresponding peak reflex changes in HR were recorded for each dose of the drug. Baroreflex sensitivity was assessed by a mean index relating changes in HR to changes in MAP, allowing a separate analysis of gain for reflex bradycardia and reflex tachycardia. The mean index was expressed as beats per minute per millimeter of mercury, as described previously [7, 8, 18].

Time domain analysis consisted of calculating mean pulse interval (PI) and SAP, with PI variability and SAP variability as the SD from its respective time series (three time series of 5 min for each animal). For frequency domain analysis, the same time series of PI and SAP were cubic spline-interpolated (250 Hz) and cubic spline-decimated to be equally spaced in time after linear trend removal. Power spectral density was obtained through the fast Fourier transformation. Spectral power for low-frequency (LF, 0.20–0.75 Hz) and high-frequency (HF, 0.75–4.0 Hz) bands was calculated by power spectrum density integration within each frequency bandwidth, using a customized routine (MATLAB 6.0, Mathworks). Beat-to-beat values of SAP and PI were used to estimate the cardiac baroreflex sensitivity by spectral analysis, using the *α* index for the LF band (0.20–0.75 Hz). The coherence between the PI and SAP signal variability was assessed through a cross-spectral analysis. The *α* index was calculated only when the magnitude of the squared coherence between the PI and SAP signals exceeded 0.5 (range 0–1) in the LF band. After coherence calculation, the *α* index was obtained from the square root of the ratio between PI and SAP variability in the LF two major bands [7, 19].

### Oxidative stress evaluations

After cardiovascular and autonomic assessments, the animals were pre-anesthetized with ketamine and euthanized by decapitation. Heart, kidney, and muscle (gastrocnemius) tissue were immediately removed and stored (− 80 °C) for oxidative stress measurements. They were then cut into small pieces, placed in ice-cold buffer, and homogenized in an ultra-Turrax blender with 1 g of tissue per 5 mL of 120 mmol/L KCl and 30 nmol/L phosphate buffer, pH 7.4. Homogenates were centrifuged at 600×*g* for 10 min at 4 °C. Protein was determined as described previously [20].

### Chemiluminescence

The lipid peroxidation membrane was evaluated by chemiluminescence (CL) in cardiac, renal, and muscle tissues. The chemiluminescence assay was carried out using an LKB Rack Beta liquid scintillation spectrometer 1215 (LKB Producer AB, USA) in the out-of-coincidence mode at room temperature. Supernatants were diluted in 140 mM KCl and 20 mM sodium phosphate buffer (pH 7.4) and added to glass tubes, which were placed in scintillation vials. 3 mM tert-butylhydroperoxide was added, and chemiluminescence was determined up to the maximal level of emission [21].

### Thiobarbituric acid reactive substances

For the thiobarbituric acid reactive substances (TBARS) assay, trichloroacetic acid (10%, w/v) was added to the homogenate to precipitate proteins and to acidify the samples. This mixture was then centrifuged (3000 rpm, 3 min), the protein-free sample was extracted, and thiobarbituric acid (0.67%, w/v) was added to the reaction medium. The tubes were placed in a water bath (100 °C) for 15 min. Absorbance was measured at 535 nm using a spectrophotometer. The results are expressed as micromoles per milligram of protein [22].

### Antioxidant enzymes

Superoxide dismutase (SOD) activity was measured spectrophotometrically in the heart, kidney, and muscle homogenates by rate inhibition of pyrogallol autoxidation at 420 nm [23]. Catalase (CAT) concentration was measured by monitoring the decrease in H_2_O_2_ concentration at 240 nm [24]. GPx activity was determined by monitoring NADPH oxidation spectrophotometrically at 340 nm [25].

### Statistical analysis

Data are presented as mean ± standard error of the mean (SEM). Data distribution was assessed using the Shapiro-Wilk test. Levene’s test was used to assess variance homogeneity. One-way analysis of variance (ANOVA) was used to compare the four groups, followed by the Student-Newman-Keuls post hoc test. Pearson correlation was used to study the relationship between variables (*n* = 4–6 animals/group, just for Pearson correlation). The significance level was established as *p* < 0.05.

## Results

Body weight was higher in the male than in the female groups and the FO group presented increased body weight when compared to both the FNOV and FOV groups at the end of the protocol (*p* < 0.05, Table 1).

**Table 1:** Body weight and cardiovascular and autonomic assessment in male rats (M), female in the non-ovulatory phase of the estrous cycle (FNOV), female in the ovulatory phase of the estrous cycle (FOV), and ovariectomized females (FO)

	M	FNOV	FOV	FO
**Body weight (g)**	381 ± 7	265 ± 5*	266 ± 4*	329 ± 4*^,†,‡^
**SAP (mmHg)**	134 ± 2	128 ± 2	137 ± 2^†^	141 ± 3^†^
**DAP (mmHg)**	100 ± 2	95 ± 2	106 ± 2^†^	103 ± 3^†^
**VAR-SAP (mmHg**^**2**^**)**	24.8 ± 2.1	24.8 ± 1.4	37.8 ± 9.4*^,†^	41.6 ± 4.1*^,†^
**LF-SAP (mmHg**^**2**^**)**	7.3 ± 0.7	3.9 ± 0.4*	5.1 ± 0.9	7.1 ± 0.8^†^
**VAR-PI (ms**^**2**^**)**	49.5 ± 5.8	44.5 ± 5.7	65.0 ± 10.0	65.1 ± 9.4
**RMSSD (ms)**	5.9 ± 0.5	4.8 ± 0.6	7.1 ± 0.6	6.8 ± 0.7
**% LF-PI (nu)**	26.3 ± 1.4	27.5 ± 2.1	30.7 ± 3.0	36.3 ± 2.3*^,†^
**% HF-PI (nu)**	73.7 ± 1.4	72.5 ± 2.1	69.3 ± 3.0	65.6 ± 2.3*

Data are presented as mean ± standard error of the mean. *n* = 7 animals/group

*p < 0.05 vs. M; ^†^*p* < 0.05 vs. FNOV; ^‡^*p* < 0.05 vs. FOV

*Abbreviations*: *SAP* systolic arterial pressure, *DAP* diastolic arterial pressure, *VAR-SAP* variance of systolic arterial pressure, *LF-SAP* low-frequency band of systolic arterial pressure, *VAR-PI* variance of pulse interval, *RMSSD* root mean square of successive RR interval differences, *LF-PI* low-frequency band of pulse interval, *HF-PI* high-frequency band of pulse interval

### Cardiovascular and autonomic assessment

**Fig. 1:**
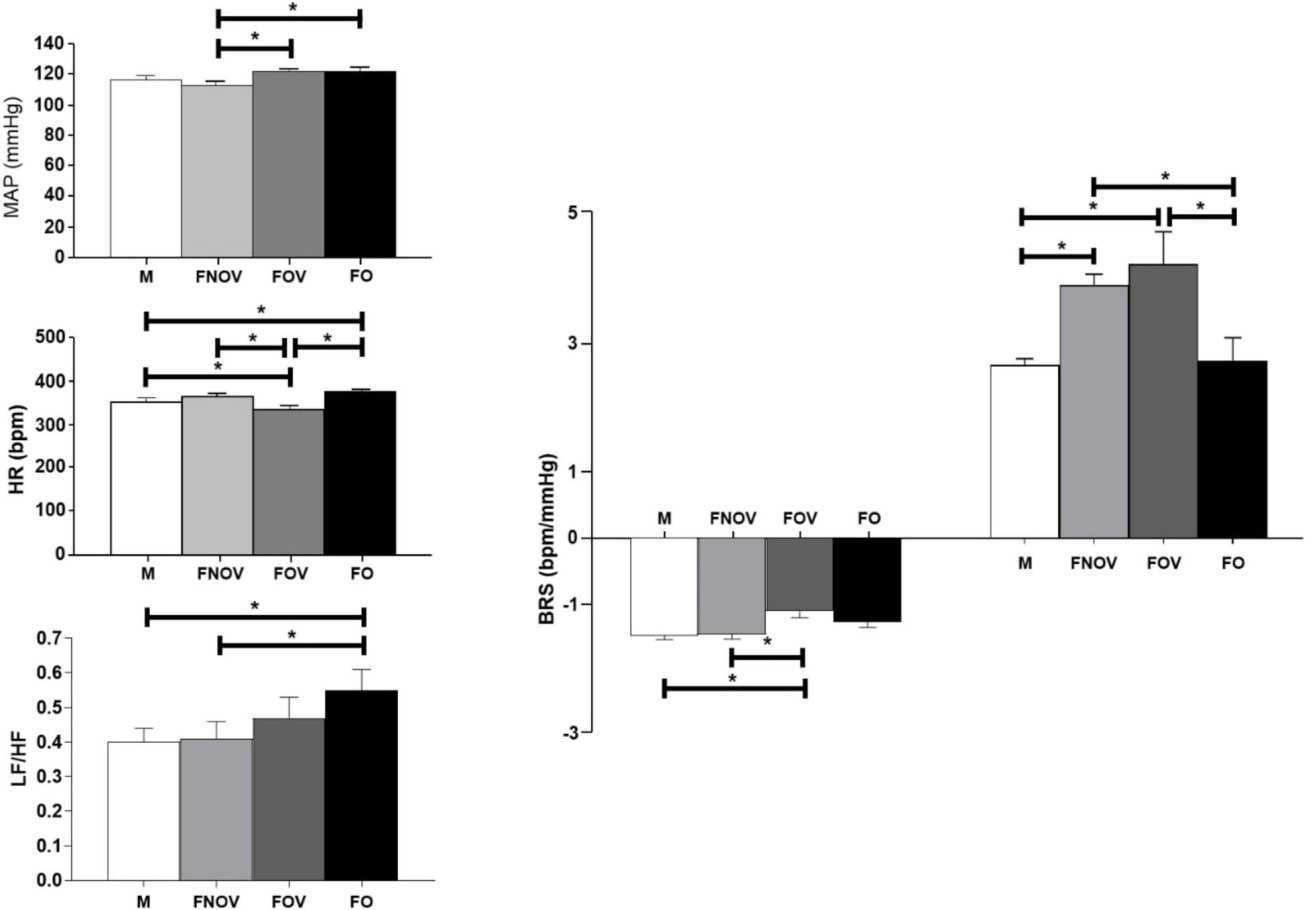
Mean arterial pressure (**a**), heart rate (**b**), cardiac sympathovagal balance represented by LF/HF ratio (**c**), and baroreflex sensitivity assessed by the index of bradycardic and tachycardic responses induced by increases and decreases in arterial pressure (**d**) in male rats (M), female in the non-ovulatory phase of the estrous cycle (FNOV), female in the ovulatory phase of the estrous cycle (FOV), and ovariectomized females (FO) (*n* = 7 animals/group). Data are presented as mean ± standard error of the mean. *p < 0.05. MAP, mean arterial pressure; LF/HF ratio, low-frequency/high-frequency ratio; BRS, baroreflex sensitivity

The hemodynamic assessment found higher values of AP (systolic, diastolic, and mean) in the FOV when compared to the FNOV group (*p* < 0.05, Table 1). In addition, deprivation of ovarian hormones (FO group) also promoted an increase in AP (MAP ~ 9 mmHg) when compared to FNOV rats (*p* < 0.05, Fig. 1). No statistical differences were observed between the M group and female groups. Basal HR was lower in FOV rats when compared to the other groups (*p* < 0.05). On the other hand, FO showed higher HR when compared to the M group (*p* < 0.05; Table 1 and Fig. 1).

In the assessment of AP variability, FNOV showed lower vascular sympathetic modulation (LF) when compared to the M group (*p* < 0.05). In addition, an increase in SAP variance (VAR-SAP) in FOV was observed when compared to M and FNOV groups. Ovarian hormone deprivation (FO group) induced an increase in VAR-SAP when compared to M and FNOV. In addition, FO showed an increase in the LF component of SAP when compared to the FNOV group (*p* < 0.05, Table 1).

There were no differences in HRV between males (M group) and the females in their reproductive phase of life (FNOV and FOV groups) in time domain analysis (*p* > 0.05). However, the FO group showed higher LF component of PI when compared to the M and FNOV groups and lower HF of PI when compared to the M group (*p* < 0.05, Table 1). Although the sympathovagal balance (LF/HF) was approximately 20% higher in FOV (0.47 ± 0.06) when compared to the M (0.40 ± 0.04) and FNOV groups (0.41 ± 0.05), no statistical differences were observed (*p* > 0.05). In turn, a higher LF/HF ratio was observed in the FO group (0.55 ± 0.06) when compared to both the M and FNOV groups (*p* < 0.05; Fig. 1).

Regarding baroreflex sensitivity, the females in the reproductive phase of life showed higher tachycardic response (FNOV 3.89 ± 0.17 and FOV 4.22 ± 0.49 bpm/mmHg) when compared to the M (2.66 ± 0.10 bpm/mmHg) and FO groups (2.73 ± 0.35 bpm/mmHg) (*p* < 0.05). The bradycardic response was reduced in FOV (1.14 ± 0.10 bpm/mmHg) when compared to both the M (1.52 ± 0.06 bpm/mmHg) and FNOV groups (1.50 ± 0.07 bpm/mmHg) (*p* < 0.05; Fig. 1).

A positive relationship involving all studied groups was found between VAR-SAP and MAP (*r* = 0.68, *p* < 0.0001) (Fig. 3).

### Oxidative stress assessment

**Fig. 2:**
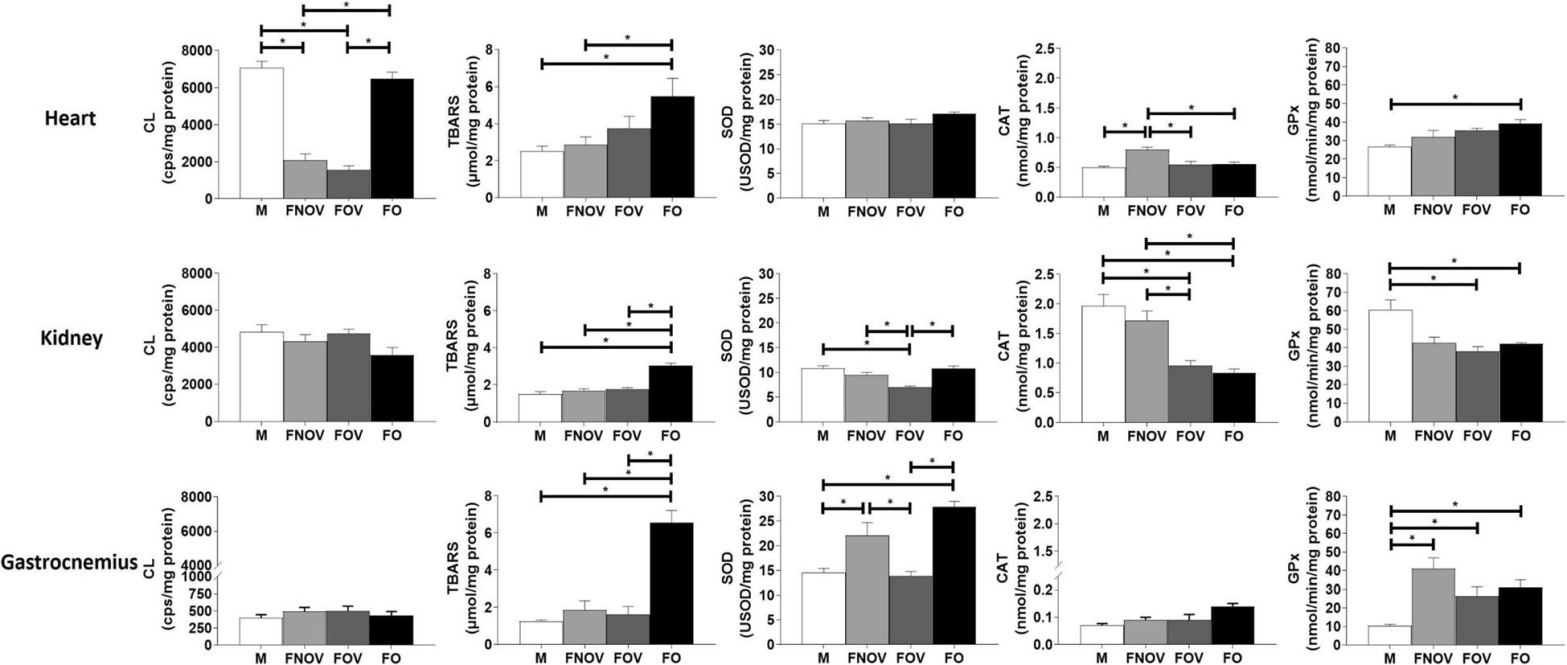
Oxidative stress assessment in cardiac, renal, and muscle tissues of male rats (M), female in the non-ovulatory phase of the estrous cycle (FNOV), female in the ovulatory phase of the estrous cycle (FOV), and ovariectomized females (FO) (*n* = 7 animals/group). Data are presented as mean ± standard error of the mean. *p < 0.05. CL, chemiluminescence; TBARS, thiobarbituric acid reactive substances; SOD, superoxide dismutase; CAT, catalase; GPx glutathione peroxidase

In their reproductive phase of life, the females presented reduced cardiac lipoperoxidation as evaluated by CL (FNOV 2084 ± 339 and FOV 1551 ± 224 cps/mg protein) when compared to male (7077 ± 339 cps/mg protein) (*p* < 0.05). Moreover, ovarian hormone deprivation promoted a higher CL (6492 ± 345 cps/mg protein) when compared to FNOV and FOV groups (*p* < 0.05); however, no statistical difference was found when compared to the M group. The FO group showed a higher cardiac lipoperoxidation as evaluated by TBARS (5.49 ± 0.95 μmol/mg protein) when compared to both the M (2.53 ± 0.27 μmol/mg protein) and FNOV groups (2.88 ± 0.41 μmol/mg protein) (*p* < 0.05). No statistical differences between male and female groups were found for SOD activity in the cardiac tissue assessments (*p* > 0.05). On the other hand, CAT activity was higher in the FNOV group when compared to other groups (*p* < 0.05). In addition, ovariectomy promoted a higher GPx between male and female groups when compared to M in this tissue (Fig. 2).

In renal tissue, CL assessment found no statistical differences between the male and all female groups (*p* > 0.05). On the other hand, ovarian hormone deprivation promoted an increase in lipid peroxidation assessed by TBARS (3.04 ± 0.13 μmol/mg protein) when compared to all other studied groups (M 1.48 ± 0.15; FNOV 1.69 ± 0.09; FOV 1.77 ± 0.07 μmol/mg protein, *p* < 0.05). Regarding SOD, FOV presented a lower SOD activity when compared to the male and female groups. In addition, FOV and ovariectomized groups demonstrated a lower CAT activity when compared to both the M and FNOV groups (*p* < 0.05). Renal tissue assessments also found a lower GPx for FOV and FO groups when compared to the M group (Fig. 2).

Similar results were found for the male and all female groups in the CL assessment in the muscle. In addition, ovariectomy induced an increase in TBARS (6.57 ± 0.65 μmol/mg protein) when compared to both male (1.26 ± 0.06 μmol/mg protein) and female groups (FNOV 1.86 ± 0.50; FOV 1.62 ± 0.43 μmol/mg protein, *p* < 0.05). SOD activity was lower in M and FOV rats when compared to FNOV and FO rats (*p* < 0.05). No statistical differences were found between male and female groups in CAT activity in the muscle tissue (*p* > 0.05), while the M group presented a lower GPx activity when compared to the female groups (*p* < 0.05, Fig. 2).

**Fig. 3.**
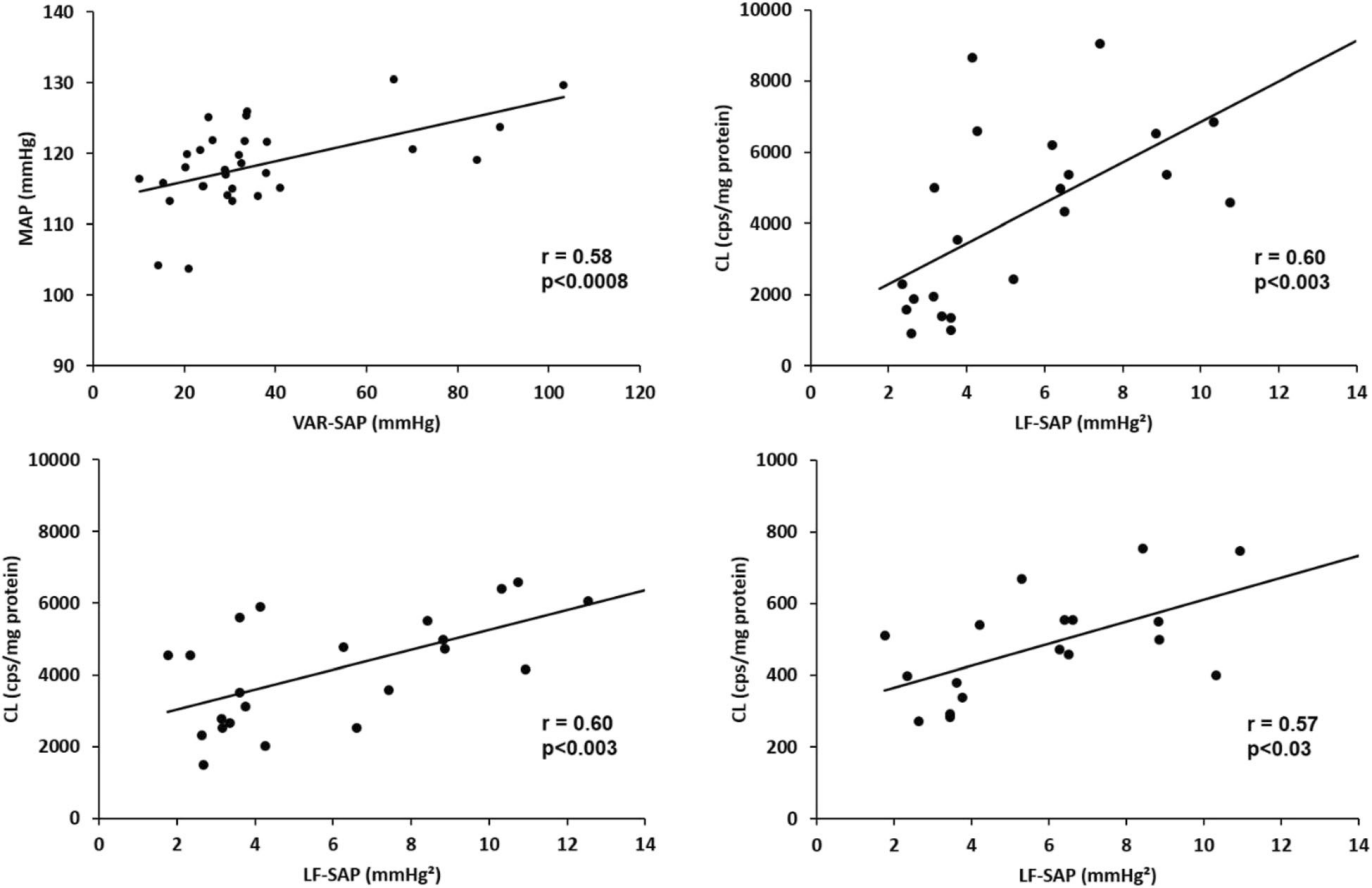
Relationship between variance of systolic arterial pressure and mean arterial pressure (**a**), sympathetic component of systolic arterial pressure and chemiluminescence in cardiac (**b**), renal (**c**), and muscle (**d**) tissues in all studied groups (*n* = 4–6 animals/group). MAP, mean arterial pressure; VAR-SAP, variance of systolic arterial pressure; CL, chemiluminescence; LF-SAP, low-frequency band of systolic arterial pressure

A positive relationship involving all studied groups was obtained between LF component of SAP variability and CL in cardiac (*r* = 0.60, *p* < 0.003), renal (*r* = 0.60, *p* < 0.003), and muscle (*r* = 0.57, *p* < 0.03) tissues (Fig. 3).

## Discussion

Our findings demonstrate the effects of ovarian hormones on cardiovascular autonomic control of circulation and on oxidative stress-induced target organ damage. Importantly, we showed that deprivation of ovarian hormones contributes to the impairment of cardiovascular control and oxidative stress, which are major risk factors for cardiovascular health in females. In addition, our data pointed to important sex differences, since a better cardiovascular autonomic modulation and oxidative stress status were found in females in the non-ovulatory phases of the estrous cycle when compared to male rats or female rats in the ovulatory phases of the estrous cycle.

The menstrual cycle is characterized by fluctuations in ovarian hormonal levels. In rats, the duration of the estrous cycle is short and the changes in the levels of estrogen and progesterone secreted by the ovarian are very intense during the different stages of the cycle. Ovulation usually occurs at the end of the estrus phase, when estrogen levels are lower [26, 27], as observed in the present study. Our findings showed that oscillation of ovarian hormones during the estrous cycle results in transitory changes in AP, as demonstrated by the increased AP in females in the ovulatory phases when compared to females in the non-ovulatory phases of the estrous cycle. The differences in AP between two phases (ovulatory and non-ovulatory) of the estrous cycle assessment in our study may be explained by the possible counterbalancing effect of estrogen on the cardiovascular system. Indeed, studies have found that progesterone concentrations were low in the estrus (ovulatory) and diestrus (non-ovulatory) phases of the estrous cycle in rats [26, 27]. Therefore, the increased AP in the FOV group is probably related to transitory reduced protective cardiovascular effects of estrogen. In fact, it has been observed that the abrupt fall in circulating estrogen levels might contribute to the rise in AP, since this hormone has direct effects on the vascular tone, RAS, and sympathetic nervous system [28]. Also, estrogen has been found to induce an increase in plasma renin activity in sheep [29]. Similarly, clinical investigation has reported elevated RAS components during luteal phase of the menstrual cycle [30], when estrogen levels are elevated.

Moreover, clinical studies have observed impaired autonomic modulation of the cardiovascular system by HRV in postmenopausal women when compared to premenopausal women [31–33]. It has been suggested that the reduced sympathetic tone in premenopausal women may contribute to help promote cardiovascular protection [34]. Data from literature suggest sympatho-inhibitory [35] and sympathoexcitatory [36] effects exerted by estrogen and progesterone, respectively. Our group has previously demonstrated that ovarian hormone deprivation induced a higher sympathetic activity to the heart when compared to control rats [9]. In line with this finding, we demonstrated in the present study that ovariectomy promoted increased LF component of PI when compared to males and to females in the non-ovulatory phases of the estrous cycle, along with lower HF component of PI when compared to male rats, indicating an increased cardiac sympathetic modulation after ovarian hormone deprivation. This finding is also supported by the cardiac sympathovagal balance observed after ovariectomy, which had a ~ 35% increase in the ovariectomized group when compared to male rats and to female rats in the non-ovulatory phases of the estrous cycle. Moreover, these autonomic changes found in ovariectomized rats were probably reflected in an increased basal HR when compared to male rats. Furthermore, females in the ovulatory phases of the estrous cycle showed increased basal HR when compared to females in the non-ovulatory phases of the estrous cycle and to males. Taken together, these data suggest that ovarian hormonal oscillation/deprivation induced cardiac autonomic changes related to basal HR alterations.

In addition, our findings demonstrated decreased baroreflex sensitivity, evaluated by bradycardic response to AP changes in females in the ovulatory phases when compared to females in the non-ovulatory phases of the estrous cycle. In fact, clinical evidence has demonstrated that ovarian hormonal fluctuations during the menstrual cycle are able to promote changes in the baroreflex regulation of sympathetic outflow. Clinical reports have also shown that estrogen may augment sympathetic baroreflex sensitivity in young women, whereas progesterone may inhibit this effect [37]. Thus, our experimental data suggest that the reduction in estrogen concentration during the estrus phase may attenuate this important cardiovascular control mechanism during the reproductive phase of life. Similarly, ovarian hormone deprivation has been found to induce increased AP and decreased baroreflex sensitivity, corroborating previous findings of our group [8, 9, 38]. Following ovariectomy, a dramatic reduction in estrogen levels has been reported, as observed in the present study, resulting in several changes in female physiological pattern. In this sense, experimental studies have shown enhanced baroreflex control of the sympathetic nervous system with subsequent AP decrease after estrogen injection into the solitary tract nucleus, rostral ventrolateral medulla, and parabrachial nucleus of ovariectomized rats [39]. This demonstrates that estrogen plays a critical role in AP changes. Interestingly, regarding the bradycardic response, our study found no differences between females in the ovulatory phase and ovariectomized. However, ovariectomized rats showed a lower baroreceptor-mediated tachycardic response when compared to the female groups, suggesting that hormonal deprivation may lead to more pronounced changes in this arm of the baroreflex. We should also like to emphasize that female rats in the different phases of the estrous cycle presented better cardiovascular autonomic control by tachycardic response than male rats. Furthermore, male and ovariectomized rats had similar tachycardic responses. Taken together, these data reinforce the positive role of ovarian hormones in cardiovascular autonomic control of circulation.

Importantly, AP variability has been largely regarded as a key marker of autonomic control, since it is associated with an increased risk of target organ damage and cardiovascular mortality [10]. In the present study, we demonstrated increased AP variability (VAR-SAP) in both ovariectomized females and females in the ovulatory phases of the estrous cycle when compared to males and to females in the non-ovulatory phases of the estrous cycle. In addition, ovarian hormone deprivation resulted in higher sympathetic modulation of SAP when compared to both male and females in the non-ovulatory phases of the estrous cycle. Moreover, we observed a positive correlation between VAR-SAP and MAP in the studied groups, demonstrating that higher AP variability was associated with higher AP resting values in the studied animals.

Further, baroreflex sensitivity has been found to be a significant predictor of cardiac event [40, 41]. A previous study of our group has demonstrated that baroreflex function impairment induced by sinoaortic denervation promotes cardiac dysfunction and collagen deposition, without changes in AP, thus strongly suggesting that isolated baroreflex dysfunction is able to modulate target organ damage [42]. Cornelli et al*.* [43] have clinically demonstrated that oxidative stress is present in more than half of the menstrual cycle, suggesting that this is a normal occurrence in women of childbearing age. In this sense, we evaluated the oxidant and antioxidant status in three important target organs, clearly demonstrating tissue-dependent responses.

Regarding oxidative stress, Massafra et al*.* [14] have not found any changes in SOD and CAT activity, despite a positive correlation between estradiol and GPx during menstrual cycle oscillations in young women. We did not observe any changes in GPx activity in female rats in our study. However, we observed decreased SOD (vs. all other groups), CAT (vs. FNOV and M), and GPx activities (vs. M) in females in the ovulatory phases of the estrous cycle in renal tissues. Moreover, CAT activity (vs. M and FOV) in cardiac tissue and GPx activities (vs. M) in muscle tissue were increased in FNOV rats. Despite these differences, lipid peroxidation was similar between male rats and female rats in the reproductive phase of life in the tissues evaluated (CL and TBARS). However, males presented a higher CL in the cardiac tissue than the females in their reproductive phase of life. As to these sex differences in CL, it should be made clear that CL is a more complex assay, with high sensitivity and specificity for lipid peroxidation, assessing the pro/antioxidant balance of tissue during the formation of peroxides induced by tert-butyl, while TBARS only measures the end products of lipoperoxidation in the sample [21]. Taken together, these findings suggest that females during the reproductive phase of life presented higher antioxidant capacity than males, mainly in their non-ovulatory phase of the estrous cycle. This may be associated with better baroreflex sensitivity and lower AP variability and, consequently, with the better cardiovascular control observed in females in the non-ovulatory phases of the estrous cycle.

Furthermore, GPx has shown to be higher in premenopausal than in postmenopausal women [12]. Using an experimental model, Gómez-Zubeldia et al*.* [44] have demonstrated increased CAT activity in plasma, but no significant change in the erythrocyte SOD activity in ovariectomized rats when compared to control rats. In the present study, we observed higher SOD and GPx in muscle and cardiac tissues, respectively, but lower CAT and GPx in renal tissue in ovariectomized females when compared to male rats. Moreover, ovariectomy reduced CAT activity in cardiac and renal tissues in females in the non-ovulatory phase of the estrous cycle. These unfavorable antioxidant changes are likely to be related to the increase in lipid peroxidation in all tissues (CL and TBARS) in ovariectomized rats, suggesting a potential role of ovarian hormones in oxidative stress levels in this model. In line with the findings of this study, our group has previously observed increased lipid peroxidation in cardiac tissue in ovariectomized rats [45]. In this sense, loss of estrogen may aggravate endothelial dysfunction and this may contribute to the onset of hypertension [46]. We would like to emphasize that we found a positive correlation between the LF component of SAP and CL in cardiac, renal, and muscle tissues, thus demonstrating that higher vascular sympathetic modulation was associated with higher levels of oxidative stress damage in target organs in the studied animals. These findings suggest that the ovulatory phase of the estrous cycle/deprivation of ovarian hormone increases phasic/chronic cardiovascular sympathetic modulation and AP variability, which, in turn, leads to changes in redox balance, probably associated with end-organ damage.

## Conclusion

Taken together, our findings point to key sex differences and the crucial role of female reproductive hormones in the cardiovascular autonomic control of circulation. Importantly, we showed that ovarian hormonal oscillation induced changes in AP along with a decrease in the baroreflex regulation of bradycardic responses and antioxidant defenses. Similarly, ovarian hormone deprivation induced autonomic impairments, probably by mediating an increase in pro-oxidants and a decrease in antioxidants, with a consequent increase in the lipoperoxidation of membranes in target tissues, resulting in AP increase.

### Perspectives and significance

The relationship between ovarian hormones and CVDs is indeed complex. While unmodifiable risk factors account for a lower percentage of mortality, hormonal changes in females have a well-established impact on women’s health. Our findings contribute to a fuller understanding of the impact of human sex differences and the crucial role played by sex hormone levels on cardiovascular autonomic control and oxidative stress in important target tissues in cardiometabolic health and disease. Since the estimates of cardiovascular mortality in the coming years are still quite challenging, our data reinforce the need for further research on the effects of ovarian hormones on cardiovascular control mechanisms in women of childbearing age and after menopause. Given the current growth of the elderly population, a fuller understanding of the impact of female sex hormones on cardiovascular control is critical, as women spend a considerable time of their lives under conditions of hormonal scarcity. As such, effective preventive and therapeutic strategies may play a major role in lowering cardiovascular risk in this population.

## Data Availability

All data are available from the corresponding author upon request.
